# Absence of endothelial α5β1 integrin triggers early onset of experimental autoimmune encephalomyelitis due to reduced vascular remodeling and compromised vascular integrity

**DOI:** 10.1186/s40478-019-0659-9

**Published:** 2019-01-24

**Authors:** Ravi Kant, Sebok K. Halder, Gregory J. Bix, Richard Milner

**Affiliations:** 1 Department of Molecular Medicine, MEM-151, The Scripps Research Institute, 10550 North Torrey Pines Road, La Jolla, CA 92037 USA; 2 Sanders-Brown Center on Aging and Department of Neurology, Neurosurgery and Neuroscience, University of Kentucky, Lexington, KY 40536 USA

**Keywords:** Endothelial, Extracellular matrix, Fibronectin, Integrin, Experimental autoimmune encephalomyelitis, Blood-brain barrier, Vascular

## Abstract

Early in the development of multiple sclerosis (MS) and its mouse model experimental autoimmune encephalomyelitis (EAE), vascular integrity is compromised. This is accompanied by a marked vascular remodeling response, though it is currently unclear whether this is an adaptive vascular repair mechanism or is part of the pathogenic process. In light of the well-described angiogenic role for the α5β1 integrin, the goal of this study was to evaluate how genetic deletion of endothelial α5 integrin (α5-EC-KO mice) impacts vascular remodeling and repair following vascular disruption during EAE pathogenesis, and how this subsequently influences clinical progression and inflammatory demyelination. Immunofluorescence staining revealed that fibronectin and α5 integrin expression were strongly upregulated on spinal cord blood vessels during the pre-symptomatic phase of EAE. Interestingly, α5-EC-KO mice showed much earlier onset and faster progression of EAE, though peak disease severity and chronic disease activity were no different from wild-type mice. At the histological level, earlier disease onset in α5-EC-KO mice correlated with accelerated vascular disruption and increased leukocyte infiltration into the spinal cord. Significantly, spinal cord blood vessels in α5-EC-KO mice showed attenuated endothelial proliferation during the pre-symptomatic phase of EAE which resulted in reduced vascular density at later time-points. Under pro-inflammatory conditions, primary cultures of α5KO brain endothelial cells showed reduced proliferation potential. These findings suggest that α5β1 integrin-mediated angiogenic remodeling represents an important repair mechanism that counteracts vascular disruption during the early stages of EAE development.

## Introduction

Multiple sclerosis (MS) is the most common neurological disease of middle-age, affecting more than 400,000 people in the United States [10, 38]. Pathologically, it is characterized as a chronic inflammatory disease in which myelin-forming oligodendrocytes are destroyed by auto-immune attack from auto-reactive T lymphocytes and monocytes, resulting in demyelination followed by degeneration of axons within the central nervous system (CNS) [11, 21]. Though auto-reactive leukocytes cause the actual damage to myelin and axons, changes in vascular properties play a central role in the initiation and maintenance of this pathology [13, 18]. Early in the disease process, the normal high integrity of CNS blood vessels, known as the blood-brain barrier (BBB) is compromised when blood vessels start to become leaky, allowing the extravasation of inflammatory leukocytes into the CNS. Within a similar time-frame, CNS blood vessels in MS patients and in an animal model of MS, experimental autoimmune encephalomyelitis (EAE), undergo a vigorous angiogenic remodeling response, culminating in increased blood vessel density [3, 15, 33]. Of note, while loss of BBB integrity has obvious deleterious consequences, it is still unclear whether the angiogenic remodeling that occurs early in MS is either part of an adaptive protective response designed to repair the damaged blood vessels and enhance the supply of oxygen and nutrients to the damaged area or is part of the pathogenic process, leading to the creation of leaky dysfunctional vessels.

Extracellular matrix (ECM) proteins play an important instructive role influencing vascular formation and stability [1, 34]. Some ECM proteins, such as laminin, are expressed at high levels during vascular differentiation and stabilization and play important roles in maintaining BBB integrity via their influence on endothelial expression of tight junction proteins [4, 25]. Conversely, other ECM proteins, such as fibronectin, and its receptor α5β1 integrin are strongly upregulated on angiogenic blood vessels in many different organs and situations, including development, inflammation and neoplasia [5, 6, 12, 16, 17, 35, 39]. We have shown that vascular formation in the CNS is associated with a developmental switch from fibronectin-mediated pathways during developmental angiogenesis to laminin-mediated pathways in the mature CNS [26]. In addition to being expressed at high levels during development, α5 integrin is strongly upregulated on remodeling blood vessels in the adult brain, as seen in mouse models of ischemic stroke, chronic mild hypoxia and MS [3, 22, 28]. Furthermore, transgenic mice with endothelial deletion of α5 integrin (α5-EC-KO mice) show delayed and reduced angiogenesis in the CNS in response to chronic mild hypoxia, highlighting an important angiogenic role for α5β1 integrin [14, 24]. In previous work, we demonstrated that in the early (pre-symptomatic) phase of EAE, blood vessels in the brain and cervical spinal cord show strong induction of fibronectin and α5β1 integrin that is associated with endothelial proliferation and a marked angiogenic response [3].

BBB disruption and a vigorous angiogenic response occur at an early stage of MS and EAE [3, 13, 18, 31, 33]. Taken with our previous work highlighting an important angiogenic role for endothelial α5β1 integrin [24], the goal of this study was to study EAE progression in mice lacking endothelial α5 integrin (α5-EC-KO mice) in order to address two key questions. First, is α5 integrin required for mediating the angiogenic response in EAE? Second, if α5 integrin is required, how does blocking angiogenesis (using α5-EC-KO mice) impact the clinical progression of EAE?

## Materials and methods

### Animals

The studies described have been reviewed and approved by The Scripps Research Institute Institutional Animal Care and Use Committee. The α5 integrin^flox/flox^ transgenic mice were a kind gift from Dr. Richard Hynes (Massachusetts Institute of Technology) and the Tie2-cre mice were obtained from Jackson Labs (Bar Harbor, ME). The generation of Tie2-Cre and α5 integrin^flox/flox^ (α5 integrin^f/f^) strains of mice and genotyping protocols have all been described previously [20, 37, 39]. All strains were backcrossed > 10 times onto the C57BL/6 background and maintained under specific pathogen-free conditions in the closed breeding colony of The Scripps Research Institute (TSRI).

### Experimental autoimmune encephalomyelitis (EAE)

EAE was performed using a protocol and materials provided by Hooke Laboratories (Lawrence, MA). Briefly, 10 week old α5-EC-KO or WT littermate control (α5^flox/flox^, Tie2-Cre negative) female mice on a C57BL6/J background were immunized subcutaneously with 200 μl of 1 mg/ml MOG_35–55_ peptide emulsified in complete Freud’s adjuvant (CFA) containing 2 mg/ml Mycobacterium tuberculosis in both the base of the tail and upper back. In addition, on days 0 and 1, mice also received an intraperitoneal injection of 200 ng pertussis toxin. In WT mice this protocol leads to robust induction of clinical EAE on days 12–14 following immunization [7, 27]. Animals were monitored daily for clinical signs and scored as follows: 0-no symptoms; 1-flaccid tail; 2-paresis of hind limbs; 3-paralysis of hind limbs; 4-quadriplegia; 5-death. Mice were euthanized at different time-points of EAE, including 0 (disease-free control), 7 (pre-symptomatic), and 16 days (symptomatic) to obtain tissue for histological studies.

### Immunohistochemistry and antibodies

Immunohistochemistry was performed on 10 μm frozen sections of cold phosphate buffer saline (PBS) perfused tissues as described previously [26]. Antibodies reactive for the following antigens were used in this study: rat monoclonals reactive to CD31 (MEC13.3), α5 integrin (5H10–27 (MFR5)), CD45 and Mac-1 (M1/70), all from BD Pharmingen (La Jolla, CA); mouse monoclonal to Ki67 from Vector Labs (Burlingame, CA), and rabbit polyclonals reactive to fibronectin (Sigma) and fibrinogen from Millipore (Temecula, CA). Fluoromyelin-red was obtained from Invitrogen. Secondary antibodies used included Cy3-conjugated anti-rat and anti-rabbit from Jackson Immunoresearch, (West Grove, PA) and Alexa Fluor 488-conjugated anti-rat and anti-mouse from Invitrogen (Carlsbad, CA).

### Image analysis

Images were acquired using a 20X objective on a Zeiss Imager M1.m. microscope. Analysis was performed specifically in the lumbar region of the spinal cord. For each antigen, four images were taken per region at 20X magnification, and a minimum of three sections per spinal cord analyzed to calculate the mean for each subject. Vascular integrity was evaluated by measuring extravascular leakage of fibrinogen, as measured by the total area of extravascular fibrinogen staining per field of view (FOV). Leukocyte infiltration indicated by levels of CD45 and Mac-1 and extent of myelination by fluoromyelin was evaluated by measuring the total area of fluorescence for each marker per FOV. Vascular expression level of α5 integrin and fibronectin was evaluated by measuring fluorescent signal intensity within a vascular mask. Endothelial proliferation was quantified by counting the number of CD31+/Ki67+ dual-positive cells per FOV. All data analysis was performed using NIH Image J software. This analysis was performed using four animals of each genotype per condition per experiment, and the results expressed as the mean ± SEM. Statistical significance was assessed using one-way or two-way analysis of variance (ANOVA) followed by Tukey’s multiple comparison post-hoc test, in which *p* < 0.05 was defined as statistically significant.

### Cell culture

Pure cultures of primary mouse brain endothelial cells (BECs) derived from α5-EC-KO or littermate control mice were prepared as previously described [29, 36]. Briefly, brains were removed from 8 week-old mice, minced, dissociated for one hour in papain and DNase I, centrifuged through 22% BSA to remove myelin, and endothelial cells cultured in endothelial cell growth media (ECGM) consisting of Hams F12, supplemented with 10% FBS, Heparin, ascorbic acid, L-glutamine, penicillin/streptomycin (all from Sigma) and endothelial cell growth supplement (ECGS) (Upstate Cell Signaling Solutions, Lake Placid, NY), on type I collagen (Sigma)-coated 6-well plates. To obtain BECs, puromycin (4 μg/ml, Alexis GmbH, Grunberg, Germany) was included in culture media between days 1–3 to remove contaminating cell types. Endothelial cell purity was > 99% as determined by CD31 in flow cytometry. For all experiments, BECs were used only for the first passage.

### Proliferation assays

Primary mouse brain endothelial cells (BECs) derived from α5-EC-KO or littermate control mice primary BEC were cultured on fibronectin-coated (10 μg/ml fibronectin (Sigma) for two hours at 37 °C) glass coverslips in the presence or absence of 10 ng/ml TNF-α (R&D, Minneapolis, MN). One day after plating, BrdU (Invitrogen, Carlsbad, CA) was added to the culture medium, and the cells incubated overnight. The next morning cells were fixed in acid/alcohol and analyzed for BrdU incorporation by incubation with a rabbit polyclonal anti-BrdU antibody (Invitrogen) for one hour followed by anti-mouse-AlexaFluor 488 secondary (Invitrogen) for one hour, then labeled with the nuclear marker Hoechst (Sigma) for 5 mins before being washed and mounted on glass slides. BrdU-positive cells were expressed as the percentage of total cells (Hoechst staining and the results presented as the mean ± SEM of four experiments.

## Results

### EAE progression is associated with upregulated expression of fibronectin and α5 integrin on spinal cord blood vessels

**Fig. 1:**
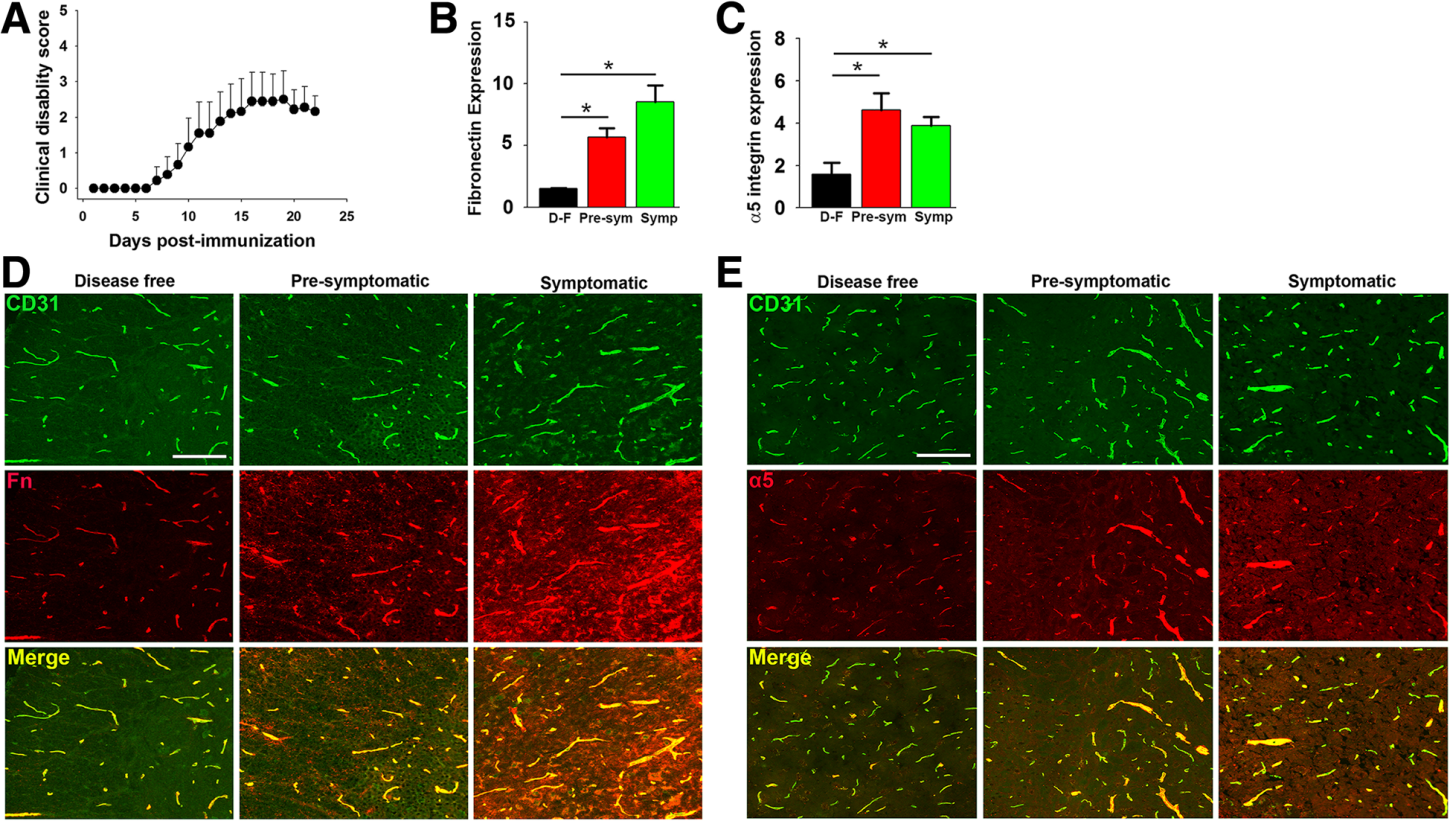
Upregulated expression of fibronectin and α5 integrin on blood vessels in the lumbar spinal cord during EAE. **a**. Time-course of increasing EAE severity following immunization. Points represent the mean ± SD (*n* = 10 mice). **b** and **c**. Quantification of fibronectin (**b**) or α5 integrin (**c**) fluorescent signal at different time-points of EAE progression. Results are expressed as the mean ± SEM (*n* = 4 mice/group). **d** and **e**. Representative examples of fibronectin and α5 integrin staining. Dual-IF was performed on frozen sections of lumbar spinal cord taken from mice that were disease-free (D-F), or in the pre-symptomatic (Pre-sym) or peak symptomatic (Symp) phase of EAE using antibodies specific for CD31 (AlexaFluor-488) and fibronectin (Fn) (Cy-3) in panel **d** or for CD31 (AlexaFluor-488) and α5 integrin (Cy-3) in panel **e**. Scale bar = 100 μm. Note that in the pre-symptomatic phase of EAE, vascular expression of both fibronectin and α5 integrin was significantly increased, and this enhanced expression level was maintained during the symptomatic phase of disease. * *p* < 0.05 vs. disease-free control

In a previous study we demonstrated that blood vessels in the brain and cervical spinal cord of mice with EAE show upregulated expression of fibronectin and α5 integrin [3]. As the earliest and most severe pathology in the EAE model occurs in the lumbar part of the spinal cord, in the current study we first wanted to determine whether this region of the spinal cord shows similar changes in vascular fibronectin and α5 integrin expression during EAE pathogenesis. To study this process, EAE was induced in 10 week old female wild-type (WT) C57BL6/J mice by immunization with MOG_35–55_ peptide, a widely-accepted model of chronic progressive MS, as previously described [3]. In keeping with findings from our lab and others [3, 7, 27], WT mice began developing clinical signs 9–12 days post-immunization (tail paralysis followed by hindlimb weakness and paralysis, and eventually quadriplegic) and disease severity gradually worsened with time (Fig. 1a). Clinical severity peaked between 15 and 21 days post-immunization and improved slightly thereafter, but mice never completely recovered. To examine how vascular expression of fibronectin and α5 integrin changes in the lumbar spinal cord during the course of EAE in WT mice, we used the endothelial marker CD31 and performed CD31/fibronectin or CD31/α5 integrin dual-immunofluorescence (IF) staining on frozen sections of lumbar spinal cord at 0, 7, and 16 days post-immunization, corresponding to disease-free control, pre-symptomatic and peak symptomatic disease, respectively. As shown in Fig. 1, under disease-free control conditions, fibronectin (Fig. 1d) and α5 integrin (Fig. 1e) were expressed at only low levels by lumbar spinal cord blood vessels, but as EAE developed, vascular expression levels of both proteins increased such that by the peak stage of EAE, fibronectin and α5 integrin were expressed at much higher levels. Quantification of fluorescent intensity (Fig. 1b) revealed that compared to disease-free conditions, vascular fibronectin expression was significantly upregulated at the pre-symptomatic stage of disease (5.75 ± 0.72 compared to 1.56 ± 0.07 fluorescent units per FOV under disease-free conditions, *p* < 0.05), and this expression level was further increased at the peak symptomatic stage of disease (8.50 ± 1.36 compared to 1.56 ± 0.07 fluorescent units per FOV under disease-free conditions, *p* < 0.05). In parallel with this upregulation of fibronectin, significant endothelial upregulation of the fibronectin receptor α5β1 integrin was detected at the pre-symptomatic stage of disease (4.56 ± 0.79 compared to 1.61 ± 0.53 fluorescent units per FOV under disease-free conditions, p < 0.05), and this enhanced expression of the α5 integrin subunit was maintained at the peak symptomatic stage of disease (3.84 ± 0.45 compared to 1.61 ± 0.53 fluorescent units per FOV under disease-free conditions, p < 0.05) (Fig. 1c).

### Genetic deletion of endothelial α5 integrin results in early onset EAE, correlating with worse neuroinflammation

**Fig. 2:**
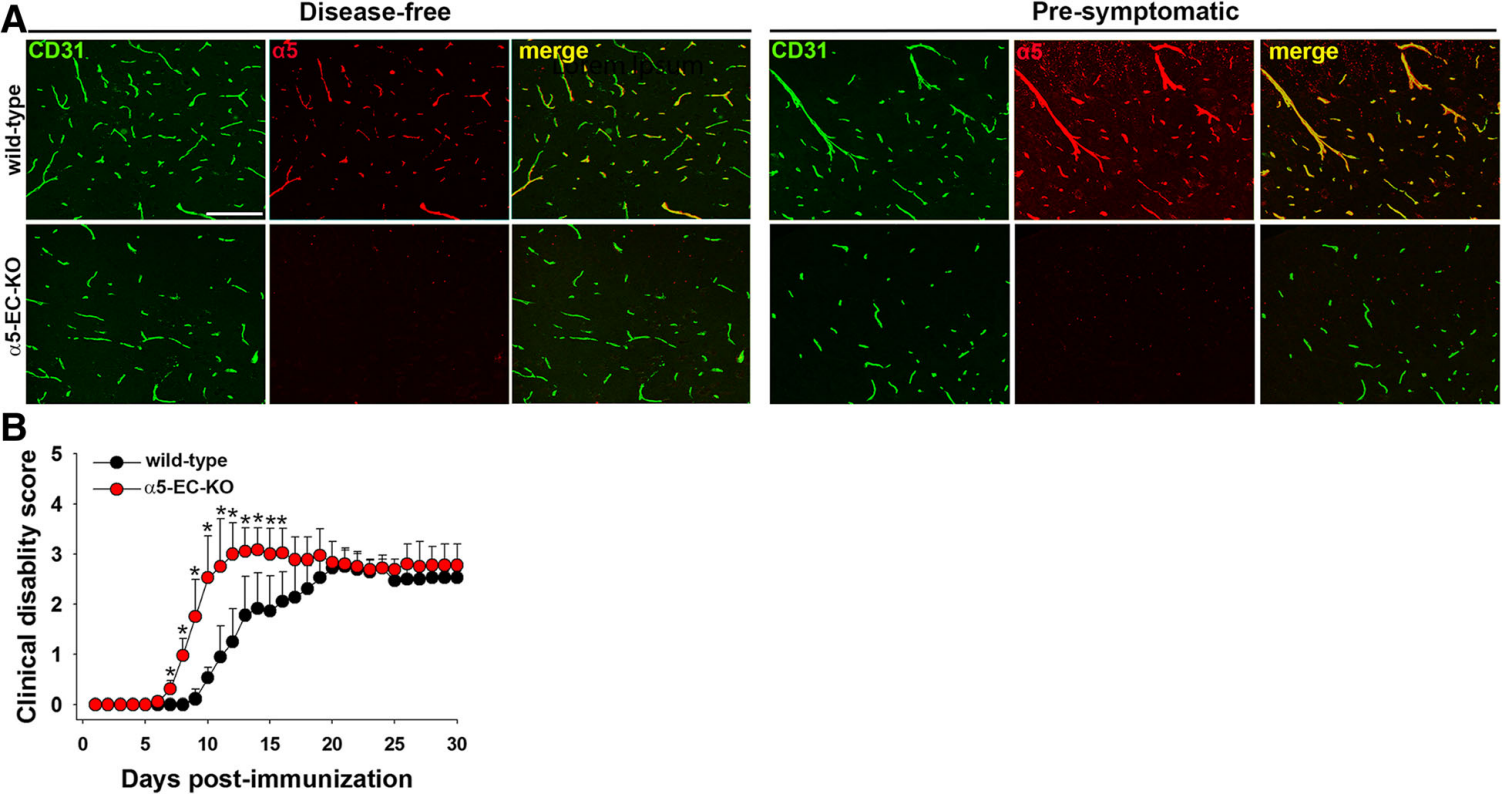
Impact of endothelial α5 integrin deletion on EAE development. **a**. Confirmation of the absence of α5 integrin expression in spinal cord endothelial cells in α5-EC-KO mice. Frozen sections of spinal cord taken from disease-free or pre-symptomatic EAE mice were processed for dual-IF for CD31 (AlexaFluor-488) and α5 integrin (Cy-3). Scale bar = 100 μm. Note that in contrast to WT spinal cord where strong upregulation of endothelial α5 integrin was observed, vessels in α5-EC-KO mice showed total lack of α5 integrin. **b**. The impact of endothelial α5 integrin deletion on clinical severity in EAE. The progression of EAE in α5-EC-KO and WT littermate control mice was evaluated by measuring clinical score on daily intervals. All points represent the mean ± SEM (*n* = 3 experiments, with 6–10 mice of each strain used per experiment). Note that compared to WT littermates, α5-EC-KO mice showed markedly earlier onset and faster progression of EAE. * *p* < 0.05 vs. WT.

To investigate the role of endothelial α5β1 integrin in modulating EAE pathogenesis, we used a Cre-Lox approach to generate mice lacking α5 integrin in endothelial cells (α5-EC-KO), by crossing floxed α5 integrin mice [37] with Tie2-Cre transgenic mice [20], as previously described [24]. Transgenic mice expressing Cre recombinase under the control of the Tie2 promoter, (Tie2-Cre; α5^f/+^) were crossed with mice in which the α5 integrin gene was floxed, i.e.; flanked by LoxP sites (α5^f/f^). From this breeding strategy, approximately 25% of the offspring were Tie2-Cre, α5^f/f^ which lacked α5 integrin expression in endothelial cells (referred to as α5-EC-KO mice). Littermate mice that had two copies of the floxed α5 integrin gene but lacking the Tie2-Cre transgene (α5^f/f^; Tie2-Cre negative) were used as wild-type (WT) controls. Importantly, α5-EC-KO mice are viable and fertile and show no obvious defects in developmental angiogenesis or vascular function under disease-free control conditions in the adult, and thus are amenable to experimental analysis [37]. To confirm that this genetic approach was effective at deleting α5 integrin from endothelial cells in these studies, we examined α5 integrin expression in sections of spinal cord taken from mice either under disease-free control conditions or at the pre-symptomatic stage of EAE. As shown in Fig. 2a, spinal cord blood vessels in WT mice maintained under disease-free conditions showed low levels of α5 integrin expression, but this expression was markedly increased at the peak of EAE disease. In contrast, α5 integrin was undetectable on spinal cord blood vessels in α5-EC-KO mice under any condition. This demonstrates that the α5 integrin gene was totally deleted from spinal cord endothelial cells within α5-EC-KO mice and it also demonstrates that endothelial cells are the major cell type expressing α5 integrin in spinal cord blood vessels [26, 28].

To investigate how genetic deletion of endothelial α5 integrin impacts the clinical progression of EAE, disease was established in 10 week old female α5-EC-KO and WT littermate mice and disease progression compared (Fig. 2b). This showed that α5-EC-KO mice developed much earlier clinical onset of EAE relative to WT littermates (mean time of onset 8.39 ± 0.86 days post-immunization vs 13.72 ± 2.51 days for WT littermates, *p* < 0.05). The mean time to reach peak disease was also much shorter in α5-EC-KO mice (11.67 ± 1.09 days vs 16.94 ± 3.00 days for WT littermates, p < 0.05). This point is well illustrated in Fig. 2b which shows that in keeping with other studies, the peak clinical score of the entire WT group was reached after approximately 20 days, but in contrast, the α5-EC-KO group reached peak clinical score after just 12 days. Thus, EAE onset and progression is significantly accelerated in α5-EC-KO mice. Interestingly however, despite these differences, by day 20 the clinical scores of α5-EC-KO and WT littermate mice were largely equivalent and remained that way until the end of the experiment (day 30). This data demonstrates that lack of endothelial α5 integrin predisposes to earlier onset and accelerated progression of EAE but has no significant impact on peak disease severity or chronic disease activity.

**Fig. 3:**
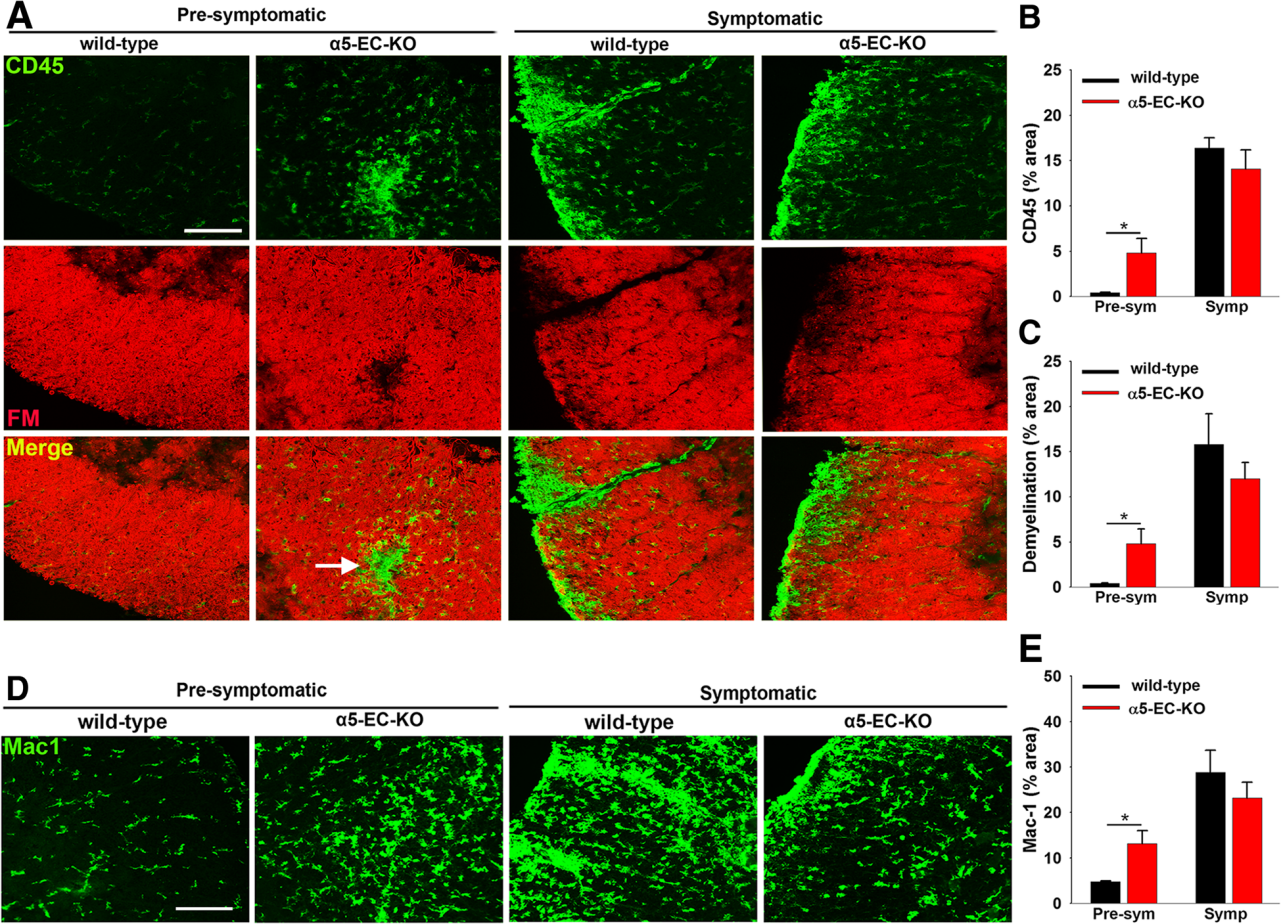
Accelerated leukocyte infiltration and demyelination in α5-EC-KO mice during EAE progression. **a** and **d**. Frozen sections of lumbar spinal cord taken from α5-EC-KO and WT littermate control mice at the pre-symptomatic and peak symptomatic phases of EAE were stained using antibodies specific for the inflammatory leukocyte marker CD45 (AlexaFluor-488) and fluoromyelin-red (FM) in panel **a** and for the leukocyte marker Mac-1 (AlexaFluor-488) in panel **d**. Quantification of CD45 (**b**), extent of demyelination (**c**) or Mac-1 (**e**) fluorescent signal at different time-points of EAE progression. Results are expressed as the mean ± SEM (*n* = 4 mice/group). Note that in the pre-symptomatic phase of EAE, α5-EC-KO mice show elevated levels of both inflammatory leukocyte markers CD45 and Mac-1 and increased levels of demyelination, as measured by a reduced fluoromyelin signal. * *p* < 0.05 vs. WT

To investigate how lack of endothelial α5 integrin impacts neuroinflammation and demyelination in this EAE model, we performed fluoromyelin/CD45 dual-IF on frozen sections of lumbar spinal cord. As shown in Fig. 3a-b, CD45 staining at the pre-symptomatic phase of EAE (7 days post-immunization) revealed that compared to WT controls, the lumbar spinal cord of α5-EC-KO mice contained significantly higher levels of CD45+ inflammatory leukocytes (4.84 ± 1.59 vs. 0.44 ± 0.05 fluorescent units per FOV, *p* < 0.05). At the same time-point (7 days), Mac-1 IF revealed increased infiltration of monocytes and activation of microglia in the spinal cord of α5-EC-KO mice as compared to WT littermates (13.12 ± 2.88 vs. 4.78 ± 0.18 fluorescent units per FOV, p < 0.05) (Fig. 3d-e). Fluoromyelin staining showed that demyelination was also more pronounced in α5-EC-KO mice relative to WT littermates at this time-point (4.62 ± 1.41 vs. 0.34 ± 0.11 fluorescent units per FOV, p < 0.05) and accumulation of CD45+ inflammatory leukocytes correlated strongly with erosion of myelin (see arrow in Fig. 3a). Interestingly however, while leukocyte infiltration and demyelination were much greater at the peak symptomatic stage of EAE (16 days post-immunization) compared to pre-symptomatic, levels between α5-EC-KO and WT littermates were not appreciably different. Thus, in this EAE model, absence of endothelial α5 integrin results in earlier onset of clinical disease, correlating with increased leukocyte infiltration and demyelination during the pre-symptomatic phase of disease, but by the symptomatic phase of EAE this difference had largely disappeared.

### Spinal cord blood vessels in α5-EC-KO mice show enhanced vascular leak at an early stage of disease

**Fig. 4:**
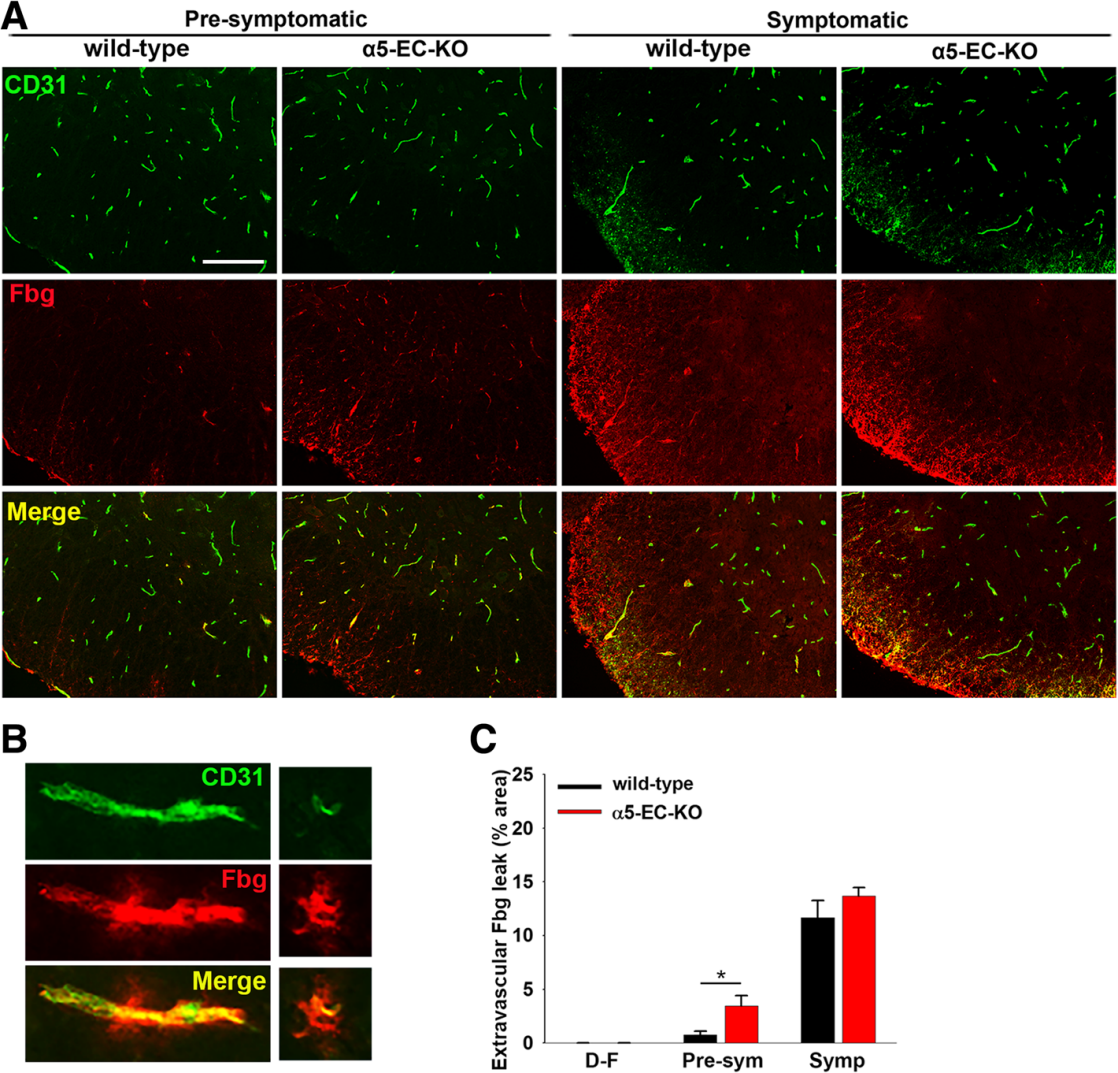
Accelerated vascular disruption in α5-EC-KO mice during EAE progression. **a**. Frozen sections of lumbar spinal cord taken from α5-EC-KO and WT littermate control mice at the pre-symptomatic and peak symptomatic phases of EAE were stained using antibodies specific for CD31 (AlexaFluor-488) and fibrinogen (Fbg) (Cy-3). Scale bar = 100 μm. **b**. High-power images of CD31+ blood vessels showing extravascular fibrinogen leak during the pre-symptomatic phase of disease. **c**. Quantification of fibrinogen leakage in α5-EC-KO vs. WT littermate mice. Results are expressed as the mean ± SEM (n = 4 mice/group). Note that at the pre-symptomatic phase of disease, while WT littermate control mice showed negligible vascular leak, α5-EC-KO mice showed obvious leakage. Interestingly though, by the peak symptomatic phase of disease, vascular leak between the two strains of mice was largely equivalent. * *p* < 0.05 vs. WT

As α5-EC-KO mice show earlier onset of EAE and increased levels of leukocyte infiltration and microglial/monocyte activation during the early pre-symptomatic stage of disease, we next examined whether the vascular integrity of spinal cord blood vessels was compromised in these mice. Using fibrinogen leak as a marker of vascular disruption, CD31/fibrinogen dual-IF showed that under disease-free conditions, there was no vascular leak in either WT or α5-EC-KO mice. However, during the pre-symptomatic (7 days post-immunization) phase of disease, while negligible extravascular leak of fibrinogen (Fbg) was detected in WT littermate control mice, α5-EC-KO mice showed obvious leak at this time-point (Fig. 4a). Quantification revealed that 7 days post-immunization, fibrinogen leak was significantly greater in α5-EC-KO mice compared to WT littermate controls (3.44 ± 0.94 compared to 0.78 ± 0.42 fluorescent units per FOV, *p* < 0.05), though interestingly, later during the peak symptomatic phase of disease (16 days post-immunization), vascular leak of fibrinogen in α5-EC-KO mice and WT littermate controls was largely equivalent (Fig. 4b).

### The pre-symptomatic angiogenic response is markedly attenuated in α5-EC-KO mice

**Fig. 5:**
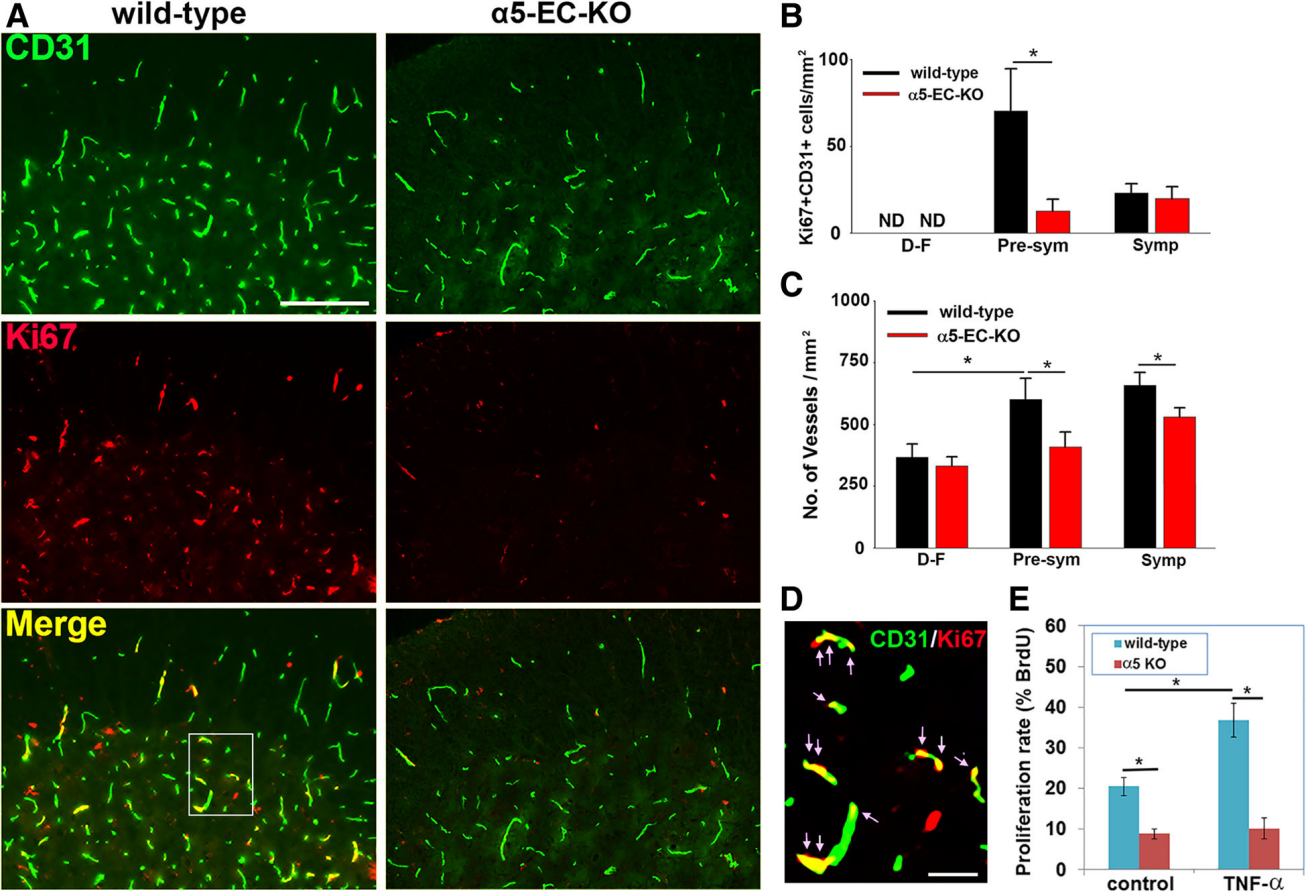
Diminished angiogenic remodeling in α5-EC-KO mice during the pre-symptomatic phase of EAE. **a**. Frozen sections of lumbar spinal cord taken from α5-EC-KO and WT littermate control mice at the pre-symptomatic phase of EAE were stained with antibodies specific for CD31 (AlexaFluor-488) and the cell proliferation marker Ki67 (Cy-3). Scale bar = 100 μm. **b** and **c**. Quantification of endothelial cell proliferation (**b**) and vascular density (**c**) in α5-EC-KO vs. WT littermate mice in disease-free (abbreviated to D-F in panels **b** and **c**), pre-symptomatic and symptomatic EAE conditions. Results are expressed as the mean ± SEM (n = 4 mice/group). Note that WT control mice showed robust endothelial proliferation during the pre-symptomatic phase of EAE, but this response was markedly reduced in α5-EC-KO mice. At the symptomatic phase, endothelial proliferation was much lower and similar in the two strains. Furthermore, in WT mice, endothelial proliferation resulted in enhanced vessel density compared to disease-free conditions, but this increase was blunted in α5-EC-KO mice. **d**. High power image of CD31/Ki67 dual-IF showing multiple proliferating endothelial cells (arrows) in WT mice at the pre-symptomatic phase of EAE. Scale bar = 25 μm. * p < 0.05 vs. WT. **e**. Quantification of proliferation of α5 integrin null and WT brain endothelial cells (BECs). Results are expressed as the mean ± SEM of 4 separate experiments. Note that TNF-α promoted proliferation of WT BECs but α5 integrin null BECs showed reduced proliferation rates and were largely unresponsive to TNF-α. * p < 0.05

In a previous study, we showed that in the pre-symptomatic phase of EAE, CNS blood vessels launch a strong vascular remodeling response that involves active endothelial proliferation leading to increased vascularity [3]. In light of our finding that endothelial α5β1 integrin plays an important angiogenic role, driving endothelial proliferation during mild hypoxia [24], we next investigated whether lack of this integrin might be stunting vascular remodeling/repair during EAE progression. To examine this, we performed CD31/Ki67 dual-IF on frozen sections of lumbar spinal cord taken from mice at different stages of EAE. This showed that in the pre-symptomatic phase of EAE, the time window during which most endothelial proliferation occurs [3], compared to disease-free control conditions (negligible endothelial proliferation), spinal cords of WT littermate mice contained numerous dual-positive CD31+/Ki67+ cells per FOV (Fig. 5a and d). However, in α5-EC-KO mice, the number of dual-positive CD31+/Ki67+ cells in the spinal cords of pre-symptomatic mice was markedly reduced (12.75 ± 6.75 vs. 70.55 ± 24.10 CD31+/Ki67+ dual-positive cells/mm^2^ in WT littermate controls, *p* < 0.05) (Fig. 5a and b). Later, during the symptomatic phase of EAE, endothelial proliferation had fallen to much lower levels with no obvious difference between the WT and α5-EC-KO strains. In keeping with the strong angiogenic response during the pre-symptomatic phase of EAE, WT mice showed a significant increase in blood vessel density, both at the pre-symptomatic (601.65 ± 85.70 compared to 366.20 ± 55.56 CD31+ vessels/mm^2^ under disease-free conditions, *p* < 0.05) and symptomatic (656.56 ± 50.50 compared to 366.20 ± 55.56 CD31+ vessels/mm^2^ under disease-free conditions, p < 0.05) phases of disease. Significantly, the impaired endothelial proliferation response observed in α5-EC-KO mice during the pre-symptomatic phase resulted in marked reduction in spinal cord blood vessel density compared to WT littermates, both at the pre-symptomatic (408.90 ± 60.50 compared to 601.65 ± 85.70 CD31+ vessels/mm^2^ in WT littermate controls, *p* < 0.05) and symptomatic phases of EAE (525.25 ± 40.40 compared to 656.56 ± 50.50 CD31+ vessels/mm^2^ in WT littermate controls, *p* < 0.05).

### Under pro-inflammatory conditions, α5 integrin null brain endothelial cells showed reduced proliferation

Following on from our observation that in EAE-affected mice, spinal cord blood vessels in α5-EC-KO mice contained fewer proliferating endothelial cells than WT mice, we wanted to test directly whether absence of α5 integrin impacts endothelial proliferation in a pro-inflammatory environment. To examine this, we isolated primary brain endothelial cells (BECs) from α5-EC-KO and WT littermate mice and cultured them on fibronectin for 24 h, at which point TNF-α was added to mimic inflammatory conditions and a BrdU incorporation assay was performed. As shown in Fig. 5e, TNF-α significantly enhanced the proliferation of WT BECs (36.8 ± 4.2 vs. 20.4 ± 2.3% under control conditions, p < 0.05) but α5 integrin null BECs showed much lower proliferation rates (8.8 ± 1.2 vs. 20.4 ± 2.3% for WT BECs under control conditions, p < 0.05) and were largely unresponsive to TNF-α (10.1 ± 2.5 vs. 8.8 ± 1.2% under control conditions, NS). These in vitro results support our in vivo observations and are consistent with the idea that endothelial α5β1 integrin confers vasculoprotection during EAE progression, in part by promoting endothelial proliferation and vascular repair.

## Discussion

At an early stage of the demyelinating disease MS or the animal model EAE, BBB disruption plays a central role in disease pathogenesis by affording leukocyte entry into the CNS [13, 18]. In previous studies we described a strong angiogenic response during the early pre-symptomatic phase of EAE that is associated with upregulated expression of α5β1 integrin on cerebral blood vessels [3]. As endothelial α5β1 integrin plays an important angiogenic role in a number of tissues including the CNS [1, 16, 24], the goal of this study was to determine how genetic deletion of endothelial α5 integrin (using α5-EC-KO mice) affects vascular remodeling and BBB integrity in the EAE model, and how this in turn, impacts clinical progression and inflammatory demyelination in this model. Our main findings were: (i) fibronectin and α5β1 integrin are strongly upregulated on lumbar spinal cord blood vessels during the progression of EAE, consistent with our previous findings in the cervical spinal cord [3], (ii) genetic deletion of endothelial α5 integrin (α5-EC-KO mice) results in markedly earlier onset of EAE and accelerated leukocyte infiltration into the CNS, (iii) spinal cord blood vessels in α5-EC-KO mice show enhanced vascular leak during the pre-symptomatic phase of EAE, (iv) spinal cord blood vessels in α5-EC-KO mice show attenuated endothelial proliferation during the pre-symptomatic phase of EAE, resulting in reduced vascular density, and (v) under pro-inflammatory conditions, primary cultures of α5KO brain endothelial cells showed reduced proliferation potential. Taken together, these studies support the notion that α5β1 integrin plays an important protective role in promoting vascular repair, thus counteracting vascular disruption during the early phase of EAE development.

### The pros and cons of vascular remodeling in MS

Vascular remodeling occurs early in MS pathogenesis [3, 31, 33]. While there is a clear consensus that BBB disruption has deleterious consequences by facilitating leukocyte infiltration into the CNS, what is less clear is the impact of angiogenic remodeling on disease progression. One fundamental question that has yet to be answered is what is the relationship between BBB disruption and angiogenic remodeling in MS? The commonly held view is that early in MS pathogenesis, BBB integrity is compromised when inflammatory leukocytes release proteolytic enzymes such as matrix metalloproteinase (MMP)-9 which digest ECM components of the vascular basement membrane as well as tight junction proteins connecting endothelial cells tightly together, to effectively punch holes in the BBB [2, 32, 40]. As endothelial proliferation and vascular remodeling occur in a similar time-frame to BBB disruption, one interpretation is that vascular remodeling is an endogenous attempt to repair the damaged blood vessels and re-establish vascular integrity. However, an alternative explanation is that the pro-inflammatory environment present during the pre-symptomatic phase of disease stimulates a dysfunctional vascular remodeling response, leading to the formation of aberrant leaky blood vessels [15, 19, 31]. The studies we present here go some way to clarifying this relationship. We report here that the absence of the pro-angiogenic α5β1 integrin largely blocked the angiogenic response normally seen in the pre-symptomatic phase of EAE [3], and that this angiogenic blockade resulted in accelerated loss of vascular integrity, worse inflammation, and faster progression of EAE. The strong implication of these findings is that vascular remodeling is a protective repair mechanism that counteracts vascular disruption at an early stage of EAE progression, and that α5β1 integrin is part of the molecular machinery that drives this angiogenic remodeling. Of note, these findings are at odds with the work of Roscoe et al. [31] who showed that antibody blockade of VEGF protected against EAE development, suggesting that silencing the angiogenic response protects against EAE progression. However, it should be pointed out that in addition to blocking the growth of new blood vessels, anti-VEGF therapy would also have the beneficial effect of enhancing vascular integrity, which by itself might account for the protective effect of the anti-VEGF treatment.

### The time-sensitivity of endothelial α5β1 integrin protection

One interesting finding to emerge from these studies is that although α5-EC-KO mice showed much earlier clinical onset of EAE, correlating with a deficiency in the ability of endothelial cells to proliferate and repair damaged blood vessels during the early stage of disease pathogenesis, surprisingly, once mice developed EAE, peak clinical scores and the extent of chronic disease in α5-EC-KO mice were essentially no different from WT littermates. What could account for this apparent time-sensitivity? Based on our previous finding that most vascular remodeling in EAE occurs in the pre-symptomatic phase [3], it follows that this phase would be most sensitive to lack of α5β1 integrin function, and that its absence at this stage would quickly lead to obvious deficits. In keeping with this idea, because relatively less angiogenesis occurs in the later phases of disease, it stands to reason that defects may not be so apparent or are more likely to be covered by compensatory mechanisms. On this note, we previously described strong upregulation of the alternative fibronectin receptor αvβ3 integrin on brain endothelial cells in response to mild hypoxia (8% O_2_) [23]. As this has the potential to compensate for loss of endothelial α5β1 integrin in promoting fibronectin-mediated cerebral angiogenesis, we also examined whether this might be happening in EAE and thus explain the lack of apparent defect in α5-EC-KO mice at later time points. However, in contrast to our studies of mild hypoxia, where αvβ3 is strongly upregulated by endothelial cells [23], in EAE tissue we saw no endothelial induction of the αvβ3 integrin at any time point examined (R. Kant and R. Milner, unpublished observations).

In a different model of neurological disease, we recently made the surprising observation that α5-EC-KO mice are profoundly resistant to experimental ischemic stroke [30]. Following ischemia, α5-EC-KO mice showed much smaller infarcts and this correlated closely with reduced levels of BBB disruption in this model. What could explain these contrasting findings in these two different models whereby endothelial α5 integrin appears to play a protective role in EAE but a potentially harmful one in ischemic stroke? One possible explanation could center around the time-scale of these two models: ischemic stroke is an acute event resulting in massive vascular disruption and loss of integrity that occurs within minutes-hours of the triggering event [8, 9], while in contrast, EAE is a more chronic event, in which vascular disruption continues for days-weeks. Another important factor could be the severity of vascular destruction. At the heart of the ischemic core in the stroke model, most blood vessels are destroyed and endothelial cells undergo cell death, while in the EAE model, blood vessels may become temporarily leaky but they are still viable and responsive to environmental cues, sufficient enough to launch an angiogenic repair response. Based on these important differences in the timing and severity of vascular injury in ischemic stroke and EAE, we propose that in the face of severe acute ischemic injury, vascular repair mechanisms mediated by endothelial α5β1 integrin are immediately overwhelmed and can do no practical good; instead, endothelial activation promoted by α5β1 integrin actually enhances endothelial separation and vascular leak, so silencing endothelial α5β1 integrin function may be protective. In contrast, in the face of the milder, more chronic challenge posed by EAE, endothelial remodeling events promoted by α5β1 integrin will facilitate vascular repair and protect against BBB disruption and neuroinflammation.

### Does endothelial α5β1 integrin represent a therapeutic target in MS?

Our studies show that the absence of endothelial α5 integrin in EAE leads to a defective vascular repair response, resulting in accelerated vascular disruption, exaggerated influx of inflammatory leukocytes and increased rate of demyelination. These findings suggest that factors that stimulate endothelial α5 integrin or its signaling pathways might enhance vascular repair and confer protection against neuroinflammatory demyelinating disease. In future studies we will test this idea by evaluating the protective activity of several fibronectin-derived peptides that specifically target the α5β1 integrin.

## Conclusions

The goal of this study was to evaluate how genetic deletion of endothelial α5 integrin affects vascular remodeling and repair following damage to the BBB during EAE pathogenesis, and how this subsequently impacts clinical progression and inflammatory demyelination. IF analysis showed that fibronectin and α5 integrin expression were strongly upregulated on spinal cord blood vessels during the pre-symptomatic phase of EAE. Interestingly, α5-EC-KO mice showed much earlier onset and faster progression of EAE, which at the histological level correlated with accelerated loss of vascular integrity and increased leukocyte infiltration into the spinal cord. Significantly, spinal cord blood vessels in α5-EC-KO mice showed attenuated endothelial proliferation during the pre-symptomatic phase of EAE which resulted in reduced vascular density. Taken together, our data support the concept that α5β1 integrin-mediated angiogenic remodeling represents an important endogenous vascular repair mechanism that counteracts vascular disruption during the early stages of EAE progression. In light of the potential importance of these findings to BBB integrity, aside from MS, our work may have implications for other chronic diseases that include BBB disruption as a pathogenic component, including vascular dementia and amyotrophic lateral sclerosis (ALS).

## Abbreviations

BBB: Blood-brain barrier
CFA: Complete Freund’s adjuvant
CNS: Central nervous system
Dual-IF: Dual-immunofluorescence
EAE: Experimental autoimmune encephalomyelitis
EC: Endothelial cell
ECM: Extracellular matrix
FOV: Field of view
KO: Knockout
MOG: Myelin oligodendrocyte glycoprotein
MS: Multiple sclerosis
SEM: Standard error of the mean
WT: Wild-type
